# Pharmacokinetics of Lekethromycin in Swine Following Intramuscular Administration at Different Doses with a Single Intravenous Reference Dose for Absolute Bioavailability and Matrix Comparison

**DOI:** 10.3390/vetsci13030294

**Published:** 2026-03-20

**Authors:** Qinyao Wu, Zeyu Wen, Jinyan Meng, Runlin Yu, Nuoyu Xu, Lu Zhang, Degang Zhou, Xingyuan Cao

**Affiliations:** 1 Technology Innovation Center for Food Safety Surveillance and Detection (Hainan), Sanya Institute of China Agricultural University, Sanya 572025, China; wuqinyao7711@163.com (Q.W.); 2019305010115@cau.edu.cn (N.X.); 2 National Key Laboratory of Detection for Veterinary Drug Residues and Illegal Additives, Ministry of Agriculture and Rural Affairs of the People’s Republic of China, Beijing 100193, China; wenzy1124@163.com (Z.W.); jinyanmemg00@outlook.com (J.M.); yurunliny@163.com (R.Y.); violetlily209@163.com (L.Z.); 3 Henan Pulike Biological Engineering Co., Ltd., Luoyang 471000, China; zhoudegang@pulike.com.cn

**Keywords:** lekethromycin, pharmacokinetics, swine, macrolide antibiotic

## Abstract

Lekethromycin (LKMS) is a novel macrolide veterinary antimicrobial. Its propensity for intracellular accumulation causes discrepancies between whole blood and plasma concentrations, complicating pharmacokinetic evaluations. This study compared the pharmacokinetic characteristics, dose proportionality, and bioavailability of LKMS in whole blood and plasma following intramuscular administration in pigs. Forty-two healthy pigs received LKMS via a single intravenous reference dose (5 mg/kg) for absolute bioavailability estimation or intramuscular (1, 2.5, 5, and 10 mg/kg) injection. Pharmacokinetic parameters were calculated using non-compartmental analysis, and dose proportionality was evaluated via a power model. LKMS exhibited rapid absorption and slow elimination, with a plasma half-life of 49.25 to 67.63 h. Whole blood exposure and peak concentrations were 1.5 to 3 times higher than in plasma, indicating extensive blood cell partitioning. As the intramuscular dose increased, the whole blood-to-plasma concentration ratio decreased from 2.83 to 1.15, suggesting a saturable cell uptake mechanism. Consequently, LKMS exhibited non-linear pharmacokinetics in whole blood but demonstrated linear, dose-proportional pharmacokinetics in plasma. Absolute bioavailability based on plasma ranged from 83.2% to 119.5%. Due to saturable blood cell binding, plasma is the optimal matrix for accurately evaluating LKMS systemic exposure and bioavailability in swine.

## Simple Summary

This study investigated the pharmacokinetics of lekethromycin in pigs after intramuscular administration at 1–10 mg/kg, with a single intravenous reference dose (5 mg/kg) included solely to estimate absolute bioavailability (F) and to support matrix comparison between whole blood and plasma. Forty-two healthy pigs were enrolled and housed under standardized conditions for the duration of the study. Each pig received a single administration of lekethromycin according to their assigned dosing route. Serial blood sampling was performed at scheduled time points, and concentrations in whole blood and plasma were quantified by UPLC–MS/MS. Pharmacokinetic parameters were derived with dedicated WinNolin software (version 8.3.4 Certara; Pharsight, Mountain View, CA, USA), and dose proportionality was assessed using a linear mixed-effects approach. Whole-blood exposure (1–10 mg/kg) was non-dose-proportional, consistent with saturable blood cell partitioning, whereas linear pharmacokinetics were observed in plasma. Furthermore, the study highlighted that plasma is the optimal matrix for accurately evaluating absolute bioavailability, providing a robust scientific foundation for determining the clinical dosing regimen of lekethromycin.

## 1. Introduction

Bacterial respiratory and systemic infections constitute a primary cause of economic loss and welfare compromise in the global swine industry [1,2]. Among these challenges, the Porcine Respiratory Disease Complex (PRDC) is particularly devastating. PRDC is a multifactorial disease characterized by coinfections with pathogens such as *Actinobacillus pleuropneumoniae*, *Pasteurella multocida*, and *Mycoplasma hyopneumoniae*. It imposes a substantial burden due to higher mortality rates, reduced feed conversion efficiency, and elevated veterinary costs [3,4]. Given the severity of these bacterial complications, antimicrobial therapy remains an essential component of veterinary clinical practice to ensure animal health, sustain production efficiency, and safeguard food security. Among the available antimicrobial classes, macrolide antibiotics are widely utilized in swine medicine due to their favorable safety profile, potent activity against respiratory pathogens, and distinct pharmacokinetic properties [5]. Tulathromycin, tilmicosin, tylvalosin, tildipirosin and gamithromycin are renowned for accumulating high concentrations in the respiratory tract and immune cells, attributed to their lipophilic and weakly basic nature [6,7,8,9,10]. Beyond their bacteriostatic effects, certain macrolides exhibit immunomodulatory properties, such as promoting neutrophil efferocytosis and reducing pro-inflammatory cytokine release, which may offer additional therapeutic benefits in resolving pulmonary inflammation [11,12].

To fully leverage these therapeutic advantages, intramuscular administration is a preferred route for managing acute respiratory outbreaks to ensure rapid and reliable systemic administration in clinical practice. However, once in the systemic circulation, the unique physicochemical properties of macrolides—specifically their amphiphilicity and weak basicity—facilitate extensive intracellular accumulation via ion trapping within acidic cellular compartments, such as lysosomes [13]. This characteristic typically results in significantly higher concentrations in whole blood compared to serum or plasma, as the drug partitions extensively into erythrocytes and leukocytes [14,15]. This discrepancy raises a critical methodological issue: the choice of biological matrix for pharmacokinetic analysis. While serum or plasma concentrations are traditionally used to estimate bioavailability and clearance, they may underestimate the total body burden of drugs that are heavily sequestered in blood cells [15,16]. The whole blood concentrations may better reflect the circulating drug reservoir available for distribution to target tissues, particularly for intracellular pathogens [17]. Pharmacokinetic evaluation based solely on plasma or serum concentrations, without accounting for drug partitioning into blood cells and variability in the whole blood-to-plasma concentration ratio, may underestimate or overestimate systemic exposure [18]. Consequently, the accurate characterization of systemic exposure and the appropriate choice of matrix are critical for assessing dose proportionality, predicting therapeutic efficacy, and integrating pharmacokinetic/pharmacodynamic (PK/PD) indices [15,19,20].

Lekethromycin (LKMS) represents a novel macrolide veterinary drug currently under development for use in pigs. Its molecular structure features three polar amine groups, conferring weak basicity (pKa > 7) and moderate lipophilicity (LogD7.4 = 2.27) [21]. These specific physicochemical properties render LKMS highly analogous to the established macrolides discussed above, theoretically making it highly susceptible to the same ion-trapping mechanism and extensive intracellular sequestration. Preliminary studies in rats have demonstrated that LKMS exhibits rapid absorption, extensive tissue distribution, and high stability in liver microsomes [21,22]. However, its pharmacokinetic behavior in the target species—swine—remains to be fully characterized. Crucially, given its structural potential for blood cell accumulation, elucidating the specific relationship between whole blood and plasma kinetics is a prerequisite for defining its true systemic exposure.

Therefore, the objectives of the present study were to (1) characterize the pharmacokinetic profile of LKMS in pigs following intramuscular administration, conducting a comparative analysis of whole blood and plasma matrices; (2) evaluate dose proportionality within the clinically relevant dose range using a robust statistical framework combining a linear mixed-effects model with a power model; and (3) determine absolute bioavailability and assess the clinical implications of matrix selection for pharmacokinetic interpretation. By clarifying the matrix-dependent pharmacokinetic behavior of LKMS in pigs, this study aims to provide a rigorous scientific basis for its rational clinical application and dosage regimen determination.

## 2. Materials and Methods

### 2.1. Chemicals and Reagents

Acetonitrile (ACN) and formic acid (FA) were provided by Thermo Fisher Scientific (Pittsburgh, PA, USA). Purified water was prepared in-house using a Milli-Q system (Merck Millipore, Burlington, MA, USA). The LKMS standard (purity ≥ 97.2%) and LKMS injection (batch number: 20230401; content: 0.5 g/10 mL) were provided by Henan Pulike Biological Engineering Co., Ltd. (Luoyang, China).

### 2.2. Experimental Animals

A total of 42 healthy ternary pigs (*n* = 21 males, 21 females, approximately 2 months old, weighing 13–15 kg) provided by the Experimental Animal Center of Pulike Biological Engineering Co., Ltd., Luoyang, China, were used in the experiments. All animals were acclimatized in a controlled environment for two weeks prior to the experiment. Animals were fed a standard commercial swine diet. Water was available ad libitum. Pigs were fasted for 12 h prior to dosing and feeding was resumed 6 h after dosing, while water remained available throughout the study. All procedures were approved by the Animal Care and Use Committee of China Agricultural University (Approval No. AW30306202-2-04; Beijing, China).

### 2.3. Drug Administration and Study Design

A randomized, multi-dose, parallel-group design was employed for drug administration. The 42 healthy ternary pigs were randomly allocated into five groups: an intravenous (IV) injection group consisting of 10 pigs, and four intramuscular (IM) injection groups consisting of 8 pigs each. The dosing regimens were as follows: Group A (IV) received LKMS injection intravenously at 5 mg/kg; Groups B-E (IM) received LKMS injection intramuscularly at 1, 2.5, 5, and 10 mg/kg, respectively. The dosing regimens are shown in Table 1.

Approximately 5 mL of blood samples were collected at 0 h and at 0.083, 0.25, 0.5, 0.75, 1, 2, 3, 6, 8, 12, 24, 48, 72, 96, 120, 144, 192, 240, 312, and 360 h post-administration. The total volume of blood drawn (~60 mL over 24 h) was maintained strictly within safe limits for 13–15 kg pigs (typically < 10% of total blood volume), and all sampling procedures were conducted under veterinary supervision. At each scheduled time point, the collected blood samples were collected into anticoagulant tubes for whole-blood analysis and for plasma preparation. Whole blood samples were stored at −20 °C until analysis. Plasma was obtained by centrifugation (4000 rpm, 10 min) and stored at −20 °C until analysis.

### 2.4. Preparation of Standards

An appropriate amount of the standard substance was weighed, and 1 mg/mL stock solutions were prepared separately. The stock solution was diluted stepwise with 0.1% formic acid in water. The working solution of LKMS standard was prepared in the concentrations range of 5–1000 ng/mL. In addition, quality control samples (QCs) were prepared in blank matrix (whole blood and plasma) at 10, 20, 400, and 800 ng/mL.

### 2.5. UPLC-MS/MS Conditions

LKMS concentrations were determined by a UPLC–MS/MS system operated in positive ESI mode. Detection was performed in MRM using the transition m/z 402.9 → 115.7. Chromatographic separation was achieved on an ACQUITY UPLC BEH C18 column (2.1 mm × 100 mm, 1.7 μm) maintained at 40 °C at a flow rate of 0.3 mL/min. Mobile phase A was 0.1% formic acid in water and mobile phase B was 0.1% formic acid in acetonitrile. The gradient program was programmed as follows: 5–100% of B (0–1.5 min), 95% of B (1.5–2.5 min), 100–50% of B (2.5–3.0 min), and 50–5% of B (3.0–5.0 min).

### 2.6. Sample Preparation

Sample preparation relied on a protein-precipitation extraction step. Briefly, a 100 μL aliquot of the whole blood/plasma sample was precipitated with 400 μL of acetonitrile and vortexed for 5 min. After mixing, the suspension was centrifuged (12,000 rpm, 12 min, 4 °C). Subsequently, a 400 μL aliquot of the supernatant was collected and evaporated to near dryness under a gentle stream of nitrogen at room temperature. Reconstitution was carried out by adding 300 μL of an aqueous 0.1% formic acid solution to the residue. After vortexing and thorough mixing, the sample was filtered through a 0.22 μm microporous filter membrane. A 10 μL aliquot of the final extract was then analyzed via UPLC-MS/MS to determine the LKMS content.

### 2.7. Method Validation

Calibration curves of LKMS were prepared over three consecutive days and demonstrated satisfactory linearity within the concentration range of 5 to 1000 ng/mL (r^2^ > 0.99). Method selectivity was assessed by analyzing six blank samples obtained from different pigs, prepared according to the sample preparation protocol described above; the blank matrices showed no interfering peaks at the retention time of LKMS. The results indicate that the intra- and inter-batch precision of QCs remained within ±15%, while the lower limit of quantification (LLOQ) samples precision remained within ±20%.

QC samples were kept at 25 °C and in the autosampler at 8 °C for 24 h to assess short-term and autosampler stability, respectively, and both conditions met the stability criteria. The drug was proven to be stable under multiple conditions, including repeated freeze–thaw cycles between −20 °C and room temperature (with each cycle lasting 12 h), and during long-term storage in blood at −20 °C for 30 days. Furthermore, evaluation of the matrix effect indicated that its impact on the experimental results was negligible (less than 15%). Dilution integrity was assessed using 10-fold and 1000-fold dilution factors, demonstrating that the recovery rates of the diluted blood samples ranged from 85% to 115%, which fully met the acceptance criteria.The method validation parameters are shown in Table 2.

### 2.8. Data Analysis

LKMS concentrations in whole blood and plasma were quantified using the validated method. Pharmacokinetic parameters were derived by non-compartmental analysis (NCA) using Phoenix WinNonlin software (Version 8.3.4, Certara/Pharsight, Mountain View, CA, USA) and are presented as the mean ± standard deviations (SDs). The drug-time curves were plotted using Origin 2024. The relationship between dose and pharmacokinetic parameters was analyzed employing a linear mixed-effects model. A one-way analysis of variance (ANOVA) was performed to compare the pharmacokinetic parameters across the different IM dose groups. The difference was significant when the *p*-value was smaller than 0.05.

## 3. Results

### 3.1. Pharmacokinetic Characteristics and Matrix Discrepancies

**Figure 1:**
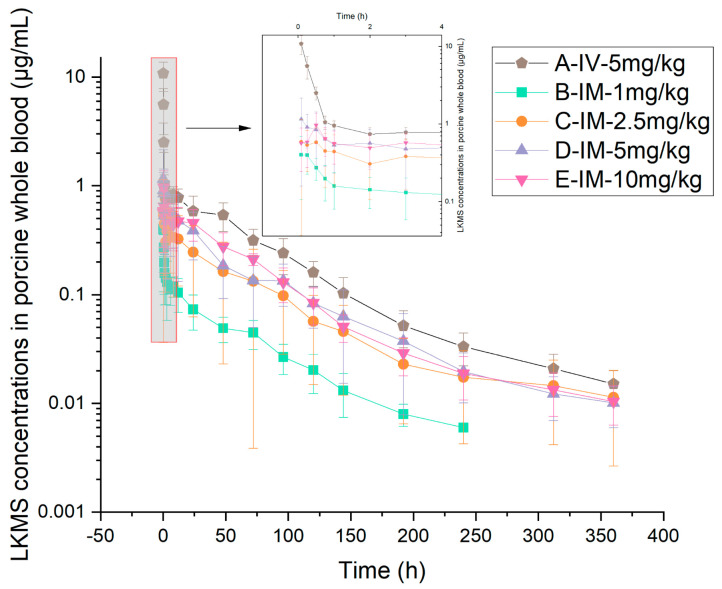
Semi-logarithmic plots of whole blood LKMS concentrations versus time after intravenous (5 mg/kg) and intramuscular (1, 2.5, 5, and 10 mg/kg) administration in pigs (mean ± SD; *n* = 10 for IV group, *n* = 8 per IM group).

**Figure 2:**
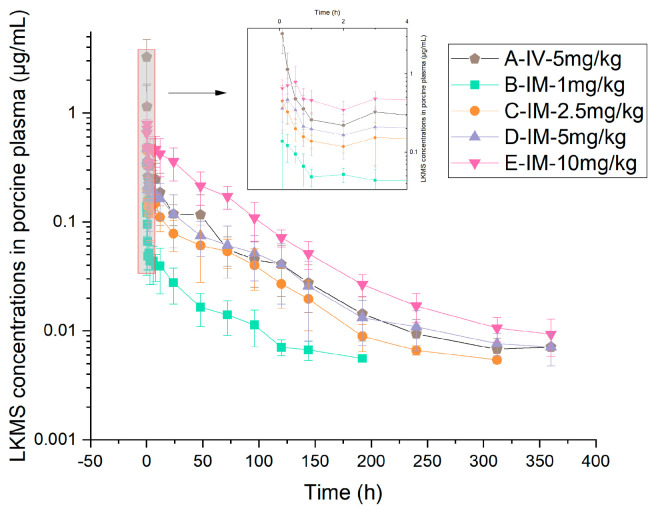
Semi−logarithmic plasma concentration–time curves of LKMS following intravenous (5 mg/kg) and intramuscular (1, 2.5, 5, and 10 mg/kg) administration in pigs (mean ± SD; *n* = 10 for IV group, *n* = 8 per IM group).

The mean concentration–time profiles and principal pharmacokinetic parameters of LKMS in pig whole blood and plasma across the five treatment groups (Table 1) are presented in Figure 1 and Figure 2, and Table 3 and Table 4.

Following a single IV administration of 5 mg/kg, LKMS concentrations exhibited a rapid distribution phase followed by a prolonged elimination phase. Pharmacokinetic analysis revealed that the total systemic exposure in whole blood (67.87 h*μg/mL) was 4.4-fold higher than that in plasma (15.43 h*μg/mL). The mean terminal elimination half-lives (t_1/2_) in whole blood and plasma were 70.14 h and 65.20 h, respectively, while the mean residence times (MRT_last_) were 66.17 h and 60.44 h, respectively.

After single IM administrations (1–10 mg/kg), LKMS was absorbed rapidly, with the mean time to maximum concentration (T_max_) occurring within 0.5 h across all dose cohorts. In all IM groups, the drug concentrations and corresponding systemic exposures in whole blood consistently exceeded those in plasma; specifically, the area under the concentration–time curve (AUC_last_) and peak concentration (C_max_) in whole blood were 1.5- to 3-fold greater than those in plasma. The drug demonstrated slow elimination kinetics in both matrices, with t_1/2_ ranging from 43.43 to 70.53 h in whole blood and 49.25 to 67.63 h in plasma. Collectively, these findings indicate that LKMS exhibits a prolonged elimination profile in swine following IM administration across varying doses.

### 3.2. Blood-to-Plasma Concentration Ratio

**Figure 3:**
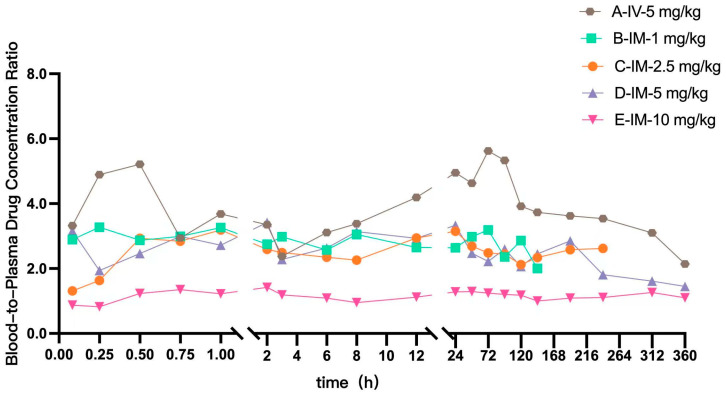
Time-course profiles of LKMS whole blood-to-plasma concentration ratios across different administration routes and dose groups (*n* = 10 for IV group, *n* = 8 per IM group).

Figure 3 illustrates the temporal distribution of LKMS whole blood-to-plasma concentration ratios across different dose groups. Following IV administration (5 mg/kg), the mean whole blood-to-plasma ratio was 3.85; for IM administrations at 1.0, 2.5, 5.0, and 10.0 mg/kg, the ratios were 2.83, 2.50, 2.53, and 1.15, respectively. These results indicate a substantial accumulation of LKMS in whole blood compared to plasma at low-to-medium doses. As the IM dose escalated, the whole blood-to-plasma ratio exhibited a pronounced dose-dependent decline, approaching unity (1.15) at the highest dose (10 mg/kg). This trend strongly suggests a saturable uptake mechanism for LKMS partitioning into blood cellular components.

### 3.3. Dose Proportionality Analysis

**Figure 4:**
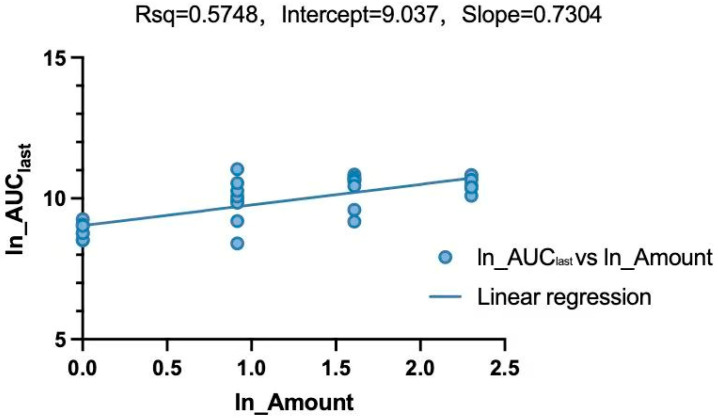
Linear regression analysis of ln(AUC_last_) against ln(Amount) following intramuscular administration (whole blood).

**Figure 5:**
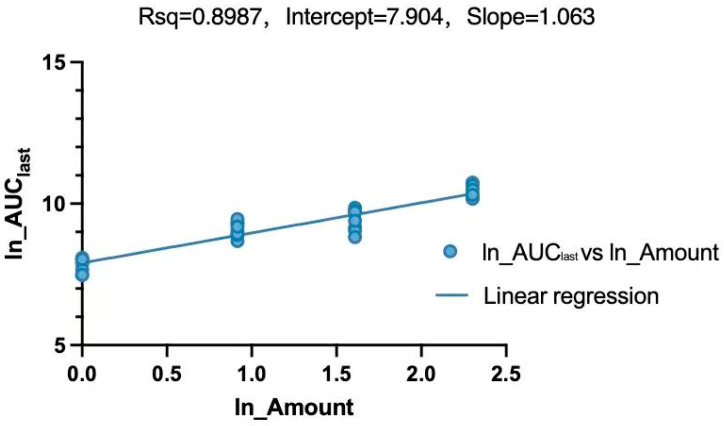
Linear regression analysis of ln(AUC_last_) against ln(Amount) following intramuscular administration (plasma).

A linear mixed-effects model combined with a power model was employed to evaluate the dose proportionality of LKMS following IM administration within the 1–10 mg/kg range (Figure 4 and Figure 5 and Table 5). A power model was applied to assess the association between the parameter (AUC_last_) and dose (Equation (1)). Dose proportionality is confirmed if the 90% confidence interval (CI) of the scaling exponent β falls within the prespecified acceptance criteria defined by Formula (2). In this study, with the dose ratio *r* = 10, the reference range for β in both whole blood and plasma is 0.70–1.30.

(1) Ln(Parameter)=α+β ∗ Ln(Amount)

(2) $$\left[1+\frac{\mathrm{Ln}(\mathrm{QL})}{\mathrm{Ln}(r)},1+\frac{\mathrm{Ln}(\mathrm{QU})}{\mathrm{Ln}(r)}\right]$$

- *r*: The ratio between the highest and lowest dose levels.
- QL: Lower equivalence limit (AUC): 0.5.
- QU: Upper equivalence limit (AUC): 2.0.

Following IM administration of LKMS across the 1–10 mg/kg range, the 90% CI for the power model exponent β in whole blood (0.55–0.93) fell outside the prespecified linear reference range (0.70–1.30). This indicates a lack of dose proportionality, demonstrating that LKMS exhibits non-linear pharmacokinetics in porcine whole blood. Conversely, the 90% CI for β in plasma (0.95–1.17) fell entirely within the reference range, confirming that systemic exposure in porcine plasma increases proportionally with dose, thereby exhibiting linear pharmacokinetics within this range.

### 3.4. Absolute Bioavailability

The absolute bioavailability (F) of LKMS in pigs was calculated using the AUC_last_ values obtained from IV administration, as described in Formula (3).

F = (AUC_IM_/Dose_IM_)/(AUC_IV_/Dose_IV_) × 100% (3)

- AUC_IM_: AUClast following IM administration.
- Dose_IM_: Administered IM dose.
- AUC_IV_: AUClast following IV administration.
- Dose_IV_: Administered IV dose.

The absolute bioavailability following a single IM injection of 1, 2.5, 5, and 10 mg/kg LKMS was 83.2%, 119.5%, 85.7%, and 107.9%, respectively. These findings indicate that the drug undergoes rapid and complete absorption following IM administration. Because LKMS exhibited non-linear pharmacokinetics in porcine whole blood (as detailed in Section 3.3), the absolute bioavailability derived from whole blood concentrations lacked dose proportionality; consequently, it was excluded as a reliable metric for assessing the extent of absorption.

## 4. Discussion

This study demonstrates that LKMS, a novel veterinary macrolide antimicrobial, exhibits pharmacokinetic characteristics of rapid absorption and slow elimination in pigs. Regarding absorption, the mean absolute bioavailability following IM administration of 1–10 mg/kg LKMS was approximately 99%, achieving more complete systemic absorption compared to tulathromycin (approximately 90%) after IM or subcutaneous administration at 2.5 mg/kg [24]. In terms of elimination, the plasma elimination half-life of LKMS ranged from 49.25 to 67.63 h, which aligns with the elimination profiles of established long-acting formulations such as tulathromycin (60–140 h) [24] and tildipirosin (approximately 106 h) [25]. Importantly, the IV reference analysis indicated a relatively low systemic clearance (CL = 0.33 ± 0.10 L/h/kg) together with extensive distribution (Vz = 31.27 ± 17.23 L/kg; Vss = 25.72 ± 13.04 L/kg). These estimates support that LKMS is removed from the body slowly and distributes widely beyond the vascular space, both of which can contribute to a prolonged terminal phase and thereby a long-acting profile. Consequently, LKMS possesses the potential for long-acting efficacy to extend clinical dosing intervals, providing pharmacokinetic data to support the subsequent optimization of targeted dosing regimens by integrating target tissue exposure with PK/PD indices.

This study observed significant distribution discrepancies of LKMS across different blood matrices. Within the 1–10 mg/kg dose range, the systemic exposure (AUC_last_) and peak concentration (C_max_) of LKMS in whole blood were significantly higher than those in plasma, with mean AUC_last_ and C_max_ ratios reaching 2.38 and 2.09, respectively. This discrepancy indicates substantial blood cell partitioning and intracellular accumulation of LKMS in porcine blood. This distribution characteristic is primarily associated with the physicochemical properties and molecular structure of macrolides. Specifically, LKMS is weakly basic (pKa > 7) and moderately lipophilic (LogD_7.4_ = 2.27) [26]. Under physiological conditions, the non-ionized form of the drug readily permeates cell membranes to enter peripheral blood phagocytes (e.g., macrophages and neutrophils) and becomes protonated within the acidic lysosomes (pH 4.5–5.0), where it is retained via the “lysosomal trapping” mechanism [27,28,29,30].

Related studies suggest that weakly basic amphiphilic drugs can significantly accumulate in acidic organelles through this lysosomal trapping mechanism. Based on physicochemical properties and pharmacokinetic modeling, Derendorf et al. predicted that the theoretical concentration of such drugs within the acidic lysosomal space is substantially higher than the extracellular concentration [31]. For instance, azithromycin concentrations in polymorphonuclear leukocytes can accumulate up to 14,217 ng/mL, exceeding plasma concentrations by a factor of 1000 [32]. Roxithromycin concentrations in leukocytes can reach 34 to 40 times that of plasma [33,34]. In veterinary medicine, the AUC_last_ of tulathromycin in porcine macrophages is approximately 500 times that in plasma [24], and the AUC_last_ of tildipirosin in porcine lung tissue can reach 87 times that in plasma [25]. Furthermore, a rat pneumonia model study demonstrated significant accumulation of LKMS in macrophage-rich spleen and lung tissues, with peak spleen concentrations reaching 5930.48 μg/kg [22].

The exposure discrepancy between whole blood and plasma for LKMS observed in this study reflects its distribution reservoir effect within blood cells. Under pathological conditions, drug-loaded blood cells can chemotactically migrate to pulmonary inflammatory lesions [24,25]. Therefore, drug accumulation within blood cells may facilitate the targeted transport of LKMS to the infection site and prolong the maintenance of local exposure, providing a mechanistic explanation for its sustained effective antimicrobial action within the infection microenvironment.

Although blood cell uptake facilitates the targeted tissue distribution of LKMS, the whole blood data indicated that this process exhibits capacity-limited characteristics. Within the 1–5 mg/kg dose range, the whole blood-to-plasma concentration ratio remained at a relatively high level of 2.50 to 3.85; however, when the dose escalated to 10 mg/kg, this ratio notably decreased, approaching unity (1.15). This alteration suggests that at high exposure levels, the uptake capacity of target cells or the lysosomal accumulation space tends toward saturation. Considering the 78–91% plasma protein binding rate of LKMS previously reported in rats [21], when both cellular uptake and protein binding simultaneously approach saturation, the proportion of unbound (free) drug in the plasma increases, leading to a decline in the whole blood-to-plasma concentration ratio.

Saturation phenomena arising from similar partitioning mechanisms have been previously documented. The entry of erythromycin into polymorphonuclear leukocytes has been confirmed to follow Michaelis-Menten saturation kinetics, with an uptake affinity constant of approximately 1.6 mM, confirming the saturability of its uptake [35]. The distribution of tacrolimus in erythrocytes is highly dependent on the intracellular binding protein FKBP12: when binding sites are unsaturated, approximately 70–75% (with 25–30% remaining in the buffer) of the drug enters the erythrocytes; however, once the binding sites are saturated, the cellular uptake proportion drops significantly to approximately 30% (with the buffer residue increasing to 70%) [36].

Influenced by the aforementioned saturation mechanism, the pharmacokinetics of LKMS in whole blood (1–10 mg/kg) exhibited non-linear characteristics, as the 90% CI of the scaling exponent β (0.55–0.93) fell outside the linear reference range of 0.70–1.30. In contrast, the dose-proportionality relationship for plasma pharmacokinetic parameters was clear, with the 90% CI of the β value falling within the prespecified range, indicating that LKMS exhibits linear plasma pharmacokinetics within the 1–10 mg/kg IM dose range.

Non-linear characteristics in whole blood are also evident in other macrolide antibiotics [37,38]. When evaluating the dose-exposure relationship of a single IM administration of 2–6 mg/kg tildipirosin, the slope of its AUC_last_ approached zero (β = 0.0153), failing to demonstrate a completely linear proportional relationship in whole blood [25]. This phenomenon aligns with established population pharmacokinetic principles regarding cellular partitioning. As Koomen demonstrated in previous theoretical models, when capacity limits exist in blood cell uptake, drug concentrations in matrices containing cellular components (such as whole blood) are prone to non-linear deviations, whereas plasma or serum levels adhere more closely to dose proportionality [39].

In pharmacokinetic evaluations, the estimation of absolute bioavailability relies heavily on the assumption of linearity between dose and systemic exposure [40]. Utilizing whole blood for calculations may lead to deviations in assessing the drug’s actual absorption efficiency. Given that LKMS injection exhibits linear pharmacokinetic characteristics in porcine plasma and accurately reflects the free drug gradient driving diffusion into peripheral tissues, this study selected plasma as the appropriate matrix for evaluating systemic exposure and absolute bioavailability.

Although this study systematically evaluated the pharmacokinetic characteristics of LKMS injection and elucidated its partitioning patterns in blood matrices, a comprehensive characterization of its targeted delivery profile still requires supplementary exposure data from target tissues. Firstly, this study primarily explored tissue distribution indirectly via the exposure discrepancy between whole blood and plasma, without directly quantifying LKMS concentrations in effector target organs, such as lung tissue or bronchoalveolar lavage fluid. Secondly, the specific impact of capacity-limited blood cell partitioning at high doses on the actual drug delivery efficiency to target organs remains to be explicitly determined. Therefore, to scientifically formulate dosing regimens, subsequent research must acquire local pharmacokinetic parameters in target tissues and integrate the saturable blood cell partitioning characteristics identified in this study into physiologically based pharmacokinetic/pharmacodynamic (PBPK/PD) models. This will facilitate an objective assessment of the dynamic exposure levels of LKMS injection at the site of infection, thereby providing data-driven support for establishing clinical dosages and dosing intervals.

## 5. Conclusions

In this study, we elucidated the matrix-dependent distribution patterns and pharmacokinetic characteristics of LKMS injection in pigs. The results demonstrate that LKMS undergoes rapid absorption and slow elimination in swine, exhibiting the characteristic blood cell partitioning behavior typical of macrolide antimicrobials, which results in significant distribution discrepancies between whole blood and plasma. Specifically, driven by capacity-limited blood cell uptake, the pharmacokinetics in whole blood exhibited non-linear characteristics over the 1–10 mg/kg dose range; in contrast, plasma pharmacokinetics demonstrated a clear dose-proportional relationship. Consequently, plasma appears to be the more appropriate matrix for evaluating the systemic exposure and absolute bioavailability of LKMS.

## Figures and Tables

**Table 1:** Dosing regimens.

Groups	Route of Administration	Dose	Sex Distribution
A	Intravenous Injection	5 mg/kg	5 females/5 males
B	Intramuscular Injection	1 mg/kg	4 females/4 males
C		2.5 mg/kg	4 females/4 males
D		5 mg/kg	4 females/4 males
E		10 mg/kg	4 females/4 males

**Table 2:** Method validation parameters for the determination of LKMS in pig blood and plasma.

Matrix	Linearity Range (ng/mL)	Correlation Coefficient, R^2^	Recovery(%)	Intra-Batch Coefficient of Variation(%)	Inter-Batch Coefficient of Variation(%)
Blood	5–1000	0.99853	98.50	<5.42	<5.63
Plasma	5–1000	0.99942	101.06	<8.16	<7.28

Validation of the analytical method was performed with reference to the “Technical Guideline for Method Validation of Quantitative Analysis of Biological Samples” [23].

**Table 3:** Primary pharmacokinetic parameters of LKMS in whole blood determined by non-compartmental analysis following intravenous (5 mg/kg) and intramuscular (1, 2.5, 5, and 10 mg/kg) administration in pigs (mean ± SD; *n* = 10 for IV, *n* = 8 per IM group).

Parameter	Units	IV (5 mg/kg)	IM (1 mg/kg)	IM (2.5 mg/kg)	IM (5 mg/kg)	IM (10 mg/kg)
AUC_INF_	h*μg/mL	69.41 ± 14.71	8.00 ± 1.82	26.83 ± 18.75	36.43 ± 15.32	39.88 ± 9.75
AUC_last_	h*μg/mL	67.87 ± 14.51	7.35 ± 1.95	25.84 ± 18.04	35.44 ± 15.17	38.92 ± 9.35
CL	L/h/kg	0.08 ± 0.02	-	-	-	-
C_max_	μg/mL	-	0.46 ± 0.25	0.79 ± 0.49	1.31 ± 0.90	1.01 ± 0.37
C0	μg/mL	15.52 ±5.63	-	-	-	-
t_1/2λz_	h	70.14 ± 10.93	43.43 ± 8.19	68.76 ± 20.31	70.53 ± 12.24	64.81 ± 25.03
MRT_last_	h	66.17 ± 5.34	50.17 ± 9.95	68.01 ± 10.94	72.58 ± 13.39	63.75 ± 8.15
T_max_	h	-	0.17 ± 0.09	0.26 ± 0.21	0.22 ± 0.14	0.42 ± 0.16
Vz	L/kg	7.56 ± 1.77	-	-	-	-
Vss	L/kg	5.59 ± 1.09	-	-	-	-

Abbreviations: AUC_INF_, area under the concentration/time curve from time 0 to infinity; AUC_last_, area under the concentration/time curve from time 0 to the last point; CL, systemic clearance (total body clearance); C_max_, maximum concentration; C0, extrapolated concentration at time zero; t_1/2λz_, terminal elimination half-life; MRT_last_, mean residence time; T_max_, time to maximum concentration; Vz, volume of distribution associated with the terminal phase (apparent volume of distribution during the terminal phase); Vss, volume of distribution at steady state.

**Table 4:** Primary pharmacokinetic parameters of LKMS in plasma determined by non-compartmental analysis following intravenous (5 mg/kg) and intramuscular (1, 2.5, 5, and 10 mg/kg) administration in pigs (mean ± SD; *n* = 10 for IV, *n* = 8 per IM group).

Parameter	Units	IV (5 mg/kg)	IM (1 mg/kg)	IM (2.5 mg/kg)	IM (5 mg/kg)	IM (10 mg/kg)
AUC_INF_	h*μg/mL	16.18 ± 4.14	3.01 ± 0.60	9.75 ± 2.27	13.99 ± 4.51	34.22 ± 7.71
AUC_last_	h*μg/mL	15.43 ± 4.07	2.57 ± 0.59	9.22 ± 2.32	13.23 ± 4.60	33.29 ± 7.51
CL	L/h/kg	0.33 ±0.10	-	-	-	-
C_max_	μg/mL	-	0.16 ± 0.09	0.47 ± 0.24	0.49 ± 0.23	0.91 ± 0.38
C0	μg/mL	5.71 ± 2.53	-	-	-	-
t_1/2λz_	h	65.20 ± 26.24	50.45 ± 8.00	49.25 ± 14.49	64.30 ± 12.71	67.63 ± 7.37
MRT_last_	h	60.44 ± 11.97	47.52 ± 5.75	58.61 ± 12.90	71.58 ± 11.62	67.34 ± 4.36
T_max_	h	-	0.25 ± 0.17	0.15 ± 0.09	0.24 ± 0.13	0.37 ± 0.30
Vz	L/kg	31.27 ± 17.23	-	-	-	-
Vss	L/kg	25.72 ± 13.04	-	-	-	-
F	%	-	83.16	119.50	85.72	107.86

Abbreviations: AUC_INF_, area under the concentration/time curve from time 0 to infinity; AUC_last_, area under the concentration/time curve from time 0 to the last point; CL, systemic clearance (total body clearance); C_max_, maximum concentration; C0, extrapolated concentration at time zero; t_1/2λz_, terminal elimination half-life; MRT_last_, mean residence time; T_max_, time to maximum concentration; Vz, volume of distribution associated with the terminal phase (apparent volume of distribution during the terminal phase); Vss, volume of distribution at steady state.

**Table 5:** Linearity analysis of LKMS following intramuscular administration based on a linear mixed-effects model.

Matrix	Model	Slope Estimate	F-Statistic	*p*-Value	Lower_CI (90%)	Upper_CI (90%)
Blood	Power model	0.73			0.54	0.93
	one-way ANOVA		40.55	<0.001		
Plasma	Power model	1.06			0.95	1.17
	one-way ANOVA		266.20	<0.001		

## Data Availability

The original contributions presented in this study are included in the article. Further inquiries can be directed to the corresponding author(s).
